# Multi-level case studies in development of complex interventions: an example of the good goals intervention

**DOI:** 10.1186/1745-6215-14-S1-P5

**Published:** 2013-11-29

**Authors:** Niina Kolehmainen, Graeme MacLennan, Laura Ternent, Edward AS Duncan, Eilidh M Duncan, Stephen B Ryan, Lorna McKee, Jill Francis

**Affiliations:** 1 University of Aberdeen, Aberdeen, UK; 2 University of Stirling, Stirling, UK; 3 Newcastle University, Newcastle, UK; 4 City University London, London, UK; 5 University of Edinburgh, Edinburgh, UK

## Background

Complex interventions often target factors across multiple levels of care provision (e.g. patient, clinician, service, wider context). Multi-level case studies provide a systematic method for modelling intervention processes and effects.

## Methods

Prospective multi-level, mixed-methods (qual+quant) case studies were used to model an intervention (‘Good Goals') to improve shared goal-setting in occupational therapy. Cases were purposively sampled (n=3 services, n=46 clinicians within these, and n=588 of their patients). Good Goals was delivered to clinicians over 25 weeks; data were collected before, during, and after delivery using interviews, focus group, case note analysis, routine data, document analysis and researchers' observations. Results were synthesised within each case, then compared across cases.

## Results

Intervention uptake was influenced by mode of delivery, competing demands on clinicians' time, and service leadership; but not by geographical or resource factors. Of hypothesised process variables, changes were observed in: clinicians' confidence, perceived social norms, and service processes. Service managers and clinicians reported positive beliefs about Good Goals' effects (e.g. it helped establish a shared rationale for clinical decisions and improved interactions with patients). Clinicians' practice changed, as observed from case notes: identifying goals, odds ratio 2.4 (95% CI 1.5-3.8); agreeing goals, 3.5 (2.4-5.1); evaluating progress, 2.0 (1.1-3.5). Patients' care episodes decreased by two months [95% CI -8 to +4] across services. Cost per clinician trained ranged from £1,003 to £1,277.

## Conclusions

Multi-level case studies identified key contextual factors and process variables, and explored likely effects on outcomes, during intervention development. This facilitates cost assessment and further intervention optimisation.

